# Does the child’s health influence the caregiver’s health using the EQ-5D instruments?

**DOI:** 10.4102/sajp.v76i1.1343

**Published:** 2020-02-26

**Authors:** Janine Verstraete, Des Scott

**Affiliations:** 1 Department of Health and Rehabilitation Sciences, Division of Physiotherapy, University of Cape Town, Cape Town, South Africa

**Keywords:** proxy, children, caregivers, health-related quality of life, EQ-5D-Y, EQ-5D-3L

## Abstract

**Background:**

Health- related quality of life (HRQoL) is an important aid in medical decision making. The child’s health may influence the caregiver’s health due to their intimate relationship.

**Objectives:**

The aim of this study was to investigate the influence of the child’s health on the caregiver’s health as measured on the EuroQoL Youth and Adult instruments.

**Method:**

A sample of 50 caregivers and their acutely-ill children, aged 3–6 years, was recruited from a paediatric hospital. Each caregiver completed the EQ-5D-Y, a proxy rating of their child’s HRQoL, and the EQ-5D-3L, a self-report measure of their own HRQoL, at baseline, 24 and 48 hours. The correlation between the caregiver and the child’s health over time was established. Forward stepwise multiple regression analysis was performed to establish the relative contribution of the child’s VAS score to the caregiver’s VAS score.

**Results:**

The results indicated that the child’s and the caregiver’s VAS ratings were significantly correlated over time, with an improvement in HRQoL scores over 48 hours. The child’s proxy VAS rating accounted for 21% and 18% of the variance in the caregiver’s VAS score at baseline and 24 hours, respectively, which was higher than self-reported problems on the caregivers EQ-5D-3L dimensions.

**Conclusion:**

The health of the caregiver is reported to improve as the perceived health of the child improves. The proxy rating of the child’s health influences the caregiver’s self-reported health more than their reported problems on the EQ-5D-3L.

**Clinical implications:**

Improving the HRQoL of the child will lead to improved HRQoL in the caregiver.

## Background

Health-related quality of life (HRQoL) can be defined as the self-rated manner in which health or healthcare impacts a person’s physical, mental and social well-being (Karimi & Brazier 2016). Thus, an individual’s HRQoL should be elicited by self-report whenever possible, even from children (Eiser & Morse 2001). This is not always possible as there are those who are either too young or cognitively unaware as to how to self-report, leaving no choice but to utilise proxy report (Cremeens, Eiser & Blades 2006; Davis et al. 2007; Drotar 2004; Eiser & Morse 2001; Eiser & Varni 2013; Varni, Burwinkle & Lane 2005). There are a number of valid and reliable HRQoL measures available for proxy report in children, including the EQ-5D-Y (Wille et al. 2010), the paediatric quality of life inventory (PedsQL) (Chen et al. 2007; Varni 2015; Varni, Seid & Rode 1999), Kidscreen (Ravens-Sieberer et al. 2008a and 2008b), KINDL and Kiddy-KINDL (Niemitz et al. 2013).

Health-related quality of life measures, such as the EQ-5D, can be used in health economic calculations to determine what effect therapeutic intervention has on HRQoL. This is typically determined with cost–utility analysis (CUA). Cost–utility analysis is typically calculated from a healthcare perspective, as the ratio between the cost of a health programme or intervention and the benefit of it in terms of the number of years the patient lives in full health (Hoefman, Van Exel & Brouwer, 2013; Neumann, Goldie & Weinstein, 2000; Torrance 1997, 2006; Weinstein, Torrance & McGuire 2009). The burden of the health state is measured by the change in quality-adjusted life years (QALYs), which takes into account quality, in terms of HRQoL utility values, and the quantity, or time spent, in a specific health condition. Quality-adjusted life-years are measured on a scale between 0 (death) and 1 (full health) where the intervals on the scale are equal and losses or gains on the scale can be aggregated (Neumann et al. 2000; Weinstein et al. 2009). Cost–utility analysis can also adopt a societal perspective in which all societal costs and effects of healthcare management are included in the calculation, regardless of who experiences them (Brouwer et al. 2006; Hoefman et al. 2013). Ignoring the costs and consequences generated from a societal perspective, such as the health of the caregiver or family, leads to healthcare decisions based on economic evaluations that have not considered all of the relevant information and could lead to underestimation of the utility gained (Al-Janabi et al. 2016; Brouwer et al., 2006; Hoefman et al. 2013).

Considering this societal perspective of economic evaluation, Verstraete, Ramma and Jelsma (2018b) explored to what extent the health of the child, as measured on the Toddler and Infant Questionnaire (TANDI), influenced the caregiver’s health, as measured on the EQ-5D-3L in children aged 1–36 months (Verstraete et al. 2018b). The study concluded that the perceived health of the child influenced the reporting of the caregiver’s own general health. The influence of the child’s health was found to be greater than the caregiver’s self-reported problems with their own pain and discomfort or mobility. Furthermore, the results showed that the general health scores of children who were acutely ill were strongly associated with the caregiver’s general health score and preference-based scores (Verstraete, Jelsma & Ramma 2018a). This result was surprising as most of the literature focusses on the effects of caring for a chronically ill child and not acutely ill children.

The caregiver’s HRQoL is affected by many factors when caring for children with chronic illness, including the child’s perceived health vulnerability (Foster et al. 2010). Mental health scores of mothers of children with Rett Syndrome (Laurvick et al. 2006) and Autism Spectrum Disorder (Rizk, Pizur-Barnekow & Darragh 2011) are lower than that of the US-based norm. Similarly, caregivers of children with mental health problems (Gerkensmeyer et al. 2013) and asthma (Zhou et al. 2014) have been shown to have higher levels of depressive symptoms and anxiety, respectively. The demands of caring for children with cerebral palsy account for lower physical and psychological health of caregivers of children when compared with the general population (Brehaut et al. 2004). Similarly, in a qualitative study exploring the effects of caring for children with disabilities, caregivers reported negative physical, emotional and functional health consequences, with most caregivers admitting that they considered their own health as a lower priority, compared to that of their child (Murphy et al. 2006)

The health condition of the child is thus thought to affect the caregiver on physical, social and psychological levels. We postulate that stressors from the child will not be the only contributing factors towards a caregiver reporting a worse HRQoL as their own health and social circumstances will also contribute to their low HRQoL (Diener et al. 1999; Uchino 2009). In children who are acutely ill, we hypothesise that the child being acutely ill would influence the caregiver’s HRQoL negatively and as the child’s health improves, the caregiver’s health improves as well. We would assume that other indirect factors linked to the child being sick such as being away from work and/or family and financial stress would remain constant through the child’s admission for acute illness. Direct and indirect factors of the child’s illness on the caregiver’s health should be captured on a HRQoL measure. The measurement of HRQoL from the point of view of both the child and parent can add value to decision-making and planning within a family-centred approach to care (Eiser & Varni 2013; Landgfuf & Abetz, 1997).

The influence of the acutely ill child’s health on the caregiver’s health has not been widely researched, and as the study by Verstraete et al. (2018a) was cross-sectional, there was no evidence that improving the health of the child would in fact improve the health of the caregiver relating to an increase in the societal QALY gained. The methodology of our study collected longitudinal data to explore this phenomenon. A suggestion resulting from the previous work by Hoefman et al. (2013) included measuring the health effects of both the caregiver and the child on the same instrument to allow for the two QALYs to be aggregated for CUA (Hoefman et al. 2013). Thus, our study aims to investigate the influence of the child’s health on the caregiver’s health as measured on the EuroQoL Youth (EQ-5D-Y) and adult (EQ-5D-3L) instruments.

The objectives include examining the correlation between the health of the child and the caregiver as measured by the caregivers’ index score and the child’s standardised severity index score, the latent value; determining whether the change in the caregiver’s Visual Analogue Scale (VAS) score, over 3 days, is proportional to the change in their report of the child’s VAS score with an improvement of the child’s health condition over 3 days. It is expected that any change in the child’s health condition will proportionally improve the caregiver’s health condition. A further objective was to explore the reasons for caregivers reporting poor HRQoL on the EQ-5D-3L.

## Methods

A longitudinal, observational, analytical cohort study was used to investigate the influence of the child’s health on the caregiver’s health as measured on the EuroQoL Youth and adult instruments. Our study was conducted at a tertiary-level paediatric hospital situated in Cape Town, South Africa.

Caregivers of children aged 3–6 years admitted to the in-patient facility of the children’s hospital were included. Caregivers who were unable to read or write English were excluded as the EQ-5D-Y proxy has not been translated or validated in any other South African language. Children who were critically ill, in the intensive care unit, were excluded owing to the associated emotional factors that participation in the study would have on the caregivers. Children who had a pre-existing chronic condition or disability were further excluded. The caregiver of the child was defined as (Department of Health, Republic of South Africa 2015):

[*A*] person who factually cares for a child (*s 1 Children’s Act, 38 of 2005*; a caregiver is obliged (in terms of s 32(1)) to safeguard the child’s health, well-being and development; and to protect the child from abuse and other harms. (p. 23)

To determine the minimum sample size of caregivers and children needed to indicate a change in the overall HRQoL between the two groups with repeated testing, the sample size calculation was based on a one-way analysis of variance (ANOVA) with a root-mean-square standardised effect (RMSSE) of 0.629, which was calculated on the anticipated difference in VAS of 16 between groups, with a standard deviation (SD) of 18.9 for two groups, with a type 1 error rate of 0.05 (Scott, Ferguson & Jelsma, 2017; Verstraete et al. 2018b). A sample of 34 caregiver and child groups was computed to ensure a power of 95% for a one-way ANOVA.

### Measures

#### Background questions

The background questions were related to the child’s age, gender and relevant medical diagnosis; relationship of caregiver to child; number of caregiving hours in the last 7 days; age; medical condition; and educational level of the caregiver. Information was also gathered on the reason behind the reporting of problems on dimensions on the EQ-5D-3L as applicable.

#### EQ-5D-Y Proxy version 1

The EQ-5D-Y Proxy version 1 includes five dimensions: mobility ‘walking about’, self-care ‘looking after myself’, usual activities ‘doing usual activities’, pain and discomfort ‘having pain or discomfort’ and a general mental health dimension labelled as ‘feeling worried, sad or unhappy’ (Wille et al. 2010). Each item has three levels of report: ‘no problems’, ‘some problems’ and ‘a lot of problems’. There is a VAS, which is a vertical, graduated scale from worst imagined health state (0) to best imagined health state (100) on which the participants rate their overall health status (Ravens-Sieberer et al. 2010; Wille et al. 2010). Proxy version 1 requires the respondent to rate the child’s health as the proxy perceives it to be. Proxy version 1 has been validated in a Spanish study in children over 6 years of age (Gusi et al. 2014), and the performance has been examined in a few studies with children older than 6 years (Bray et al. 2010).

#### EQ-5D-3L

The caregiver’s HRQoL was measured using the EQ-5D-3L, an adult self-report measure assessing five dimensions of health: mobility, self-care, usual activities, pain/discomfort and anxiety/depression, and a general rating of health status on a VAS (Brooks 1996; The EuroQol Group 1990). Similar to the youth version, each of the dimensions is rated on a three-level report. The EQ-5D-3L has been used in South Africa across health conditions, and cultural and language groups (Jelsma 2010; Jelsma & Ferguson 2004; Jelsma et al. 2005; Jelsma & Ramma, 2010; Loeb et al. 2008).

### Procedure

The children and their caregivers were recruited systematically in numerical order according to ward and cubicle allocation.

The caregivers were given detailed information regarding the study and informed consent was taken, at 24 hours or later, post-admission to the acute hospital. At baseline, caregivers were asked to complete the EQ-5D-Y Proxy for their hospitalised child, and the EQ-5D-3L, rating their own HRQoL. Furthermore, they completed contextual information and were asked to give any justification behind reporting of problems on the EQ-5D-3L. Caregivers were asked to complete the EQ-5D-Y proxy and EQ-5D-3L again at 24 and 48 hours after recruitment to measure change in HRQoL.

### Data Management and analysis

The information from EQ-5D-Y Proxy, EQ-5D-3L and contextual information were entered into an Excel spreadsheet under the code allocated to each individual.

Descriptive statistics were used to record the frequencies of responses to categorical data. The preference-based score of the caregiver was calculated using the EQ-5D-3L UK Time-trade off (TTO) index (Dolan et al. 1995). The overall proxy-rated health of the child was calculated based on the EQ-5D-Y latent value, which was generated from the UK adult population using discrete choice experiment (DCE) utility (Rivero-Arias et al. 2017). The preference-based score and latent scores both give a summary score to the five dimensions on the EQ-5D-3L and EQ-5D-Y Proxy, respectively. Correlations were done to establish the relationship between the child’s VAS and latent score and the caregiver’s VAS and preference-based score. Pearson’s correlation coefficients were interpreted according to Hinkle, Wiersma and Jurs-Boston (2013) guidelines where 0.1–0.3 is low, 0.3–0.5 is moderate and 0.5–1.0 is high (Hinkle et al. 2003). A one-way ANOVA was computed to detect the difference in change of HRQoL between the health of the caregiver and the child over time. Finally, forward stepwise multiple regression analysis was performed to establish the relative contribution of the child’s VAS score to the VAS score of the caregiver. Forward stepwise regression models were developed with the VAS score of the caregiver as the dependent variable. The independent variables for the caregiver included dummy variables where the presence of reporting problems on the EQ-5D (a lot or some problems) was collapsed into one category and compared with the absence of problems on the EQ-5D-3L and the VAS score of the child.

### Ethical consideration

The study commenced after ethical approval was obtained from the University of Cape Town Human Research Ethics Committee (HREC REF: 179/2018) and permission was granted by the children’s hospital.

Each participant of the study was required to give informed consent agreeing to participate in the research before the investigation began. Minors participating in the study (children between the ages of three and six) were considered too young to give informed consent or assent, and we therefore required that their caregiver provide informed consent on their child’s behalf. It should be noted that in order to participate, it was required that only caregivers who were able to communicate in English were included in the study as the EQ-5D-Y is currently only available in English. Caregivers and children across a full range of socio-economic backgrounds were recruited and no one who met the inclusion criteria was excluded on the grounds of ethnicity, gender preference, religion or any other reason.

Personal participant information was always protected to ensure confidentiality. We were unable to avoid documenting caregiver’s or minor’s full names in the initial raw data collection as we were ethically obligated to report risk of harm to the minor if identified at any stage. Names were deleted with data entry to ensure confidentiality. All raw data were stored in a locked cupboard and all soft data were protected in a password secured folder electronically.

Only caregivers who were present at their child’s bedside were recruited, participants were not reimbursed for their transport costs to the hospital as they did not incur additional expenses.

## Results

Fifty-one caregivers agreed to participate in baseline data collection. One participant withdrew during this process and a total of 50 participants were included for data analysis. On the second and third days of data collection, seven and six children had been discharged from the hospital, respectively. The remaining participants all completed the second and third days of testing.

The majority of the caregivers were mothers (78%) with fewer fathers (6%). The mean age of the caregivers was 43 (range 19.75–63.58; SD 9.84). Because of the acute nature of their children’s illness, many of the caregivers (51.5%) spent 24 hours at their child’s bedside. Most of the caregivers were healthy (58%), with a smaller number having a health condition. The most prevalent conditions were asthma (11%), hypertension (8%) and HIV (8%). There were two caregivers who had musculoskeletal conditions and two caregivers who reported having depression.

There were slightly more male children (54%) than female children. The reported diagnosis for admission of the child was varied and included burns (24%), general surgery (20%), respiratory illness (10%), traumatic injury (10%) and other (6%), comprising cardiac conditions, infections and organ transplant. The children were acutely ill with no underlying chronic illness or disability.

### Comparison of caregiver and child’s health scores over time

There was a large significant correlation between child’s proxy-rated VAS score and the caregiver’s VAS scores for all 3 days (Table 1). There was a large, significant correlation between the utility or preference-based score for caregivers and the latent value for the child on the second day only (*r* = 0.65, *p* < 0.001) (Table 1).

**TABLE 1a:** Correlation between the scores of the child and the caregiver over three days.

EQ-5D-Y VAS and summary scores over time	EQ-5D-3L VAS 1 Pearsons *r* (*p*-value)	EQ-5D-3L VAS 2 Pearsons *r* (*p*-value)	EQ-5D-3L VAS 3 Pearsons *r* (*p*-value)
EQ-5D-Y VAS Score 1	**0.60 (< 0.001)**	0.51 (0.002)	0.24 (0.168)
EQ-5D-Y VAS Score 2	0.29 (0.088)	**0.68 (< 0.001)**	0.25 (0.56)
EQ-5D-Y VAS Score 3	−0.04 (0.825)	0.63 (< 0.001)	**0.56 (< 0.001)**

Significant *p* values are bolded.

VAS, visual analogue scale.

**TABLE 1b:** Correlation between the scores of the child and the caregiver over three days.

EQ-5D-Y VAS and summary scores over time	EQ-5D-3L Utility 1(*p*-value)	EQ-5D-3L Utility 2(*p*-value)	EQ-5D-3L Utility 3(*p*-value)
EQ-5D-Y latent value 1	0.110 (0.537)	0.52 (0.002)	0.20 (0.255)
EQ-5D-Y latent value 2	−0.002 (0.993)	**0.65 (< 0.001)**	0.24 (0.174)
EQ-5D-Y latent value 3	−0.050 (0.763)	0.52 (0.001)	0.18 (0.313)

Significant *p* values are bolded.

VAS, visual analogue scale.

**FIGURE 1:**
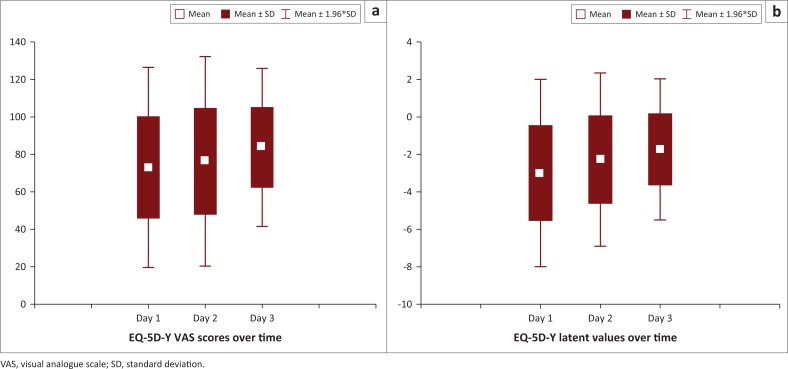
Box and whisker plot of children’s EQ-5D-Y scores over time.

The children’s proxy-rated VAS score, indicating their general health, significantly improved over time (*F* = 4.003, *p* = 0.022). The VAS score improved from 73.03 (*SD* = 27.25) on the first day to 76.27 (SD = 28.52) on the second day and 83.7 (*SD* = 21.53) on the third day (Figure 1). Similarly, the child’s proxy-rated EQ-5D-Y latent score significantly improved over time (*F* = 11.903, *p* < 0.001). The mean latent score improved from -3.0 (*SD* = 2.55) on the first day to -2.28 (*SD* = 2.40) on the second day and -1.73 (*SD* = 1.92) on the third day (Figure 1). The large SDs for the VAS and latent scores can be attributed to the heterogeneous sample of children with a large range of health conditions.

**FIGURE 2:**
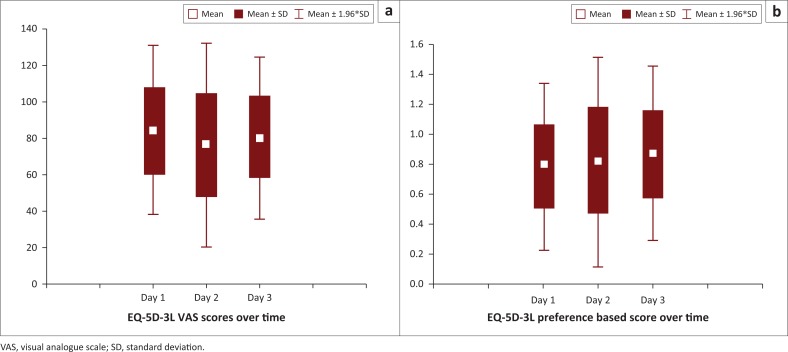
Box and whisker plot of caregivers’ EQ-5D-3L scores over time.

The mean VAS score, indicating general health, of the caregivers decreased from the first (mean = 80.86, *SD* = 22.41) to the second administration (mean = 76.03, *SD* = 28.35). There was, however, an increase in VAS scores between the first and the third administration (mean = 84.81; *SD* = 24.46). The change in VAS scores over time was not significant (*F* = 1.642, *p* = 0.201) (Figure 2). The caregivers’ EQ-5D-3L preference-based score improved over time but not significantly (*F* = 2.723; *p* = 0.073). The mean preference-based score improved from 0.75 (*SD* = 0.28) on the first day to 0.77 (*SD* = 0.38) on the second day and 0.87 (*SD* = 0.29) on the third day (Figure 2).

Caregiver’s dimension scores show that there was an increase in problems on the physical dimensions (mobility, self-care and usual activities) on the second day. There was a decrease in the reporting of pain or discomfort and anxiety or depression on the second and third days (Table 2).

**TABLE 2:** Caregivers dimension scores over the 3 days.

EQ-5D-3L Dimensions	Day 1 (*n* = 50)						Day 2 (*n* = 42)						Day 3 (*n* = 36)					
	1		2		3		1		2		3		1		2		3	
	*n*	%	*n*	%	*n*	%	*n*	%	*n*	%	*n*	%	*n*	%	*n*	%	*n*	%
Mobility	48	96	2	4	-	-	38	91	3	7	1	2	35	97	1	3	-	-
Self-care	48	96	2	4	-	-	40	96	1	2	1	2	35	97	1	3	-	-
Usual activities	42	84	5	10	3	6	35	83	2	5	5	12	31	86	3	8	2	6
Pain or discomfort	30	60	18	36	2	4	31	74	8	19	3	7	30	83	4	11	2	6
Anxiety or depression	29	58	16	32	5	10	32	76	6	14	4	10	28	78	5	14	3	8

1, no problems; 2, some problems; 3, a lot of problems.

### Influence of child’s perceived general health on the caregiver’s health

Multiple regression analysis with the caregiver’s EQ-5D-3L VAS score from day 1 as the dependent variable and dummy variables representing the different levels of the dimensions of the EQ-5D-3L and the child’s EQ-5D-Y Proxy VAS score on the first day accounted for 51% of the variance. Problems with anxiety or depression on the EQ-5D-3L (*p* = 0.004), the caregiver’s age (*p* = 0.003) and the child’s EQ-5D-Y VAS score (*p* < 0.001) all significantly influenced the caregiver’s VAS (Table 3).

**TABLE 3:** Regression analysis of the caregiver’s EQ-5D-3L visual analogue scale score on day 1.

Items	b*	Standard error of b*	*b*	Standard error of *b*	*t*(35)	*p*
Intercept	-	-	59.6	17.477	3.41	0.002
Child’s age	−0.129	0.106	−2.3	1.902	−1.22	0.230
Caregiver’s age	0.356	0.112	0.8	0.239	3.19	**0.003**
Number of caregiving hours	−0.112	0.102	−0.5	0.450	−1.10	0.280
Presence of medical condition in the caregiver	0.018	0.107	0.8	4.658	0.17	0.864
EQ-5D-3L problems with mobility	−0.100	0.240	−14.5	34.747	−0.42	0.679
EQ-5D-3L problems with self-care	−0.100	0.240	−14.5	34.747	−0.42	0.679
EQ-5D-3L problems with usual activities	0.037	0.115	2.2	6.770	0.32	0.752
EQ-5D-3L problems with pain/discomfort	−0.106	0.110	−4.6	4.691	−0.97	0.339
EQ-5D-3L problems with anxiety/depression	−0.352	0.113	−15.0	4.809	−3.12	**0.004**
Child’s EQ-5D-Y VAS score	0.399	0.109	0.3	0.090	3.65	0.001

Singificant *p*-values < 0.05 are indicated in bold.

b*, denotes standardised beta regression coefficients.

*b*, denotes non-standardised beta regression coefficients.

*n* = 48, adjusted *R*^2^ = 0.51.

VAS, visual analogue scale.

A summary of forward stepwise regression analysis indicates that the child’s proxy rating of VAS accounted for most variance (21%), and this was followed by the caregiver’s report of anxiety or depression (6%) and the caregiver’s age (12%).

Multiple regression analysis with the caregiver’s EQ-5D-3L VAS score from day 2 as the dependent variable and the caregiver’s EQ-5D-3L dimension scores and the child’s EQ-5D-Y Proxy VAS score on the second day accounted for 25% of the variance. The child’s EQ-5D-Y Proxy VAS score (*p* < 0.010) was the only factor, which significantly influenced the caregiver’s VAS score (Table 4).

**TABLE 4:** Regression analysis of the caregiver’s EQ-5D-3L visual analogue scale score on day 2.

EQ-5D-3L Dimensions	b*	Standard error of b*	*b*	Standard error of *b*	*t*(27)	*p*
Intercept	-	-	35.7	117.486	0.30	0.764
Child’s age	0.068	0.152	6.2	13.925	0.44	0.661
Caregiver’s age	−0.009	0.169	−0.1	1.933	−0.05	0.957
Number of care giving hours	0.016	0.166	3.4	36.320	0.09	0.925
Presence of medical condition in the caregiver	−0.067	0.154	−1.5	3.448	−0.44	0.666
EQ-5D-3L problems with mobility	0.037	0.245	18.2	119.557	0.15	0.880
EQ-5D-3L problems with self-care	0.100	0.242	67.8	164.702	0.41	0.684
EQ-5D-3L problems with usual activities	−0.236	0.186	−70.6	55.562	−1.27	0.214
EQ-5D-3L problems with pain/discomfort	−0.180	0.213	−44.5	52.755	−0.84	0.406
EQ-5D-3L problems with anxiety/depression	−0.088	0.205	−23.6	54.808	−0.43	0.670
EQ-5D-Y VAS score 2	0.518	0.188	1.9	0.675	2.75	**0.010**

Singificant *p*-values < 0.05 are indicated in bold.

b*, denotes standardised beta regression coefficients.

*b*, denotes non-standardised beta regression coefficients.

*n* = 38, adjusted *R*^2^ = 0.25.

A summary of forward stepwise regression analysis indicates that on the second day, the child’s perceived health as rated on the VAS accounted for 18% of the variance, which was more than the combined variance contributed by problems with usual activities (4%) and pain or discomfort (2%).

Regression analysis did not indicate any significant detraction from the caregiver’s HRQoL on the third day.

The reason for the caregiver’s reporting problems on the EQ-5D-3L dimensions is shown in Table 5.

**TABLE 5:** Reasons behind the scoring of the EQ-5D-3L questionnaire on day 1.

EQ-5D-3L Dimensions	Problems related to child	Problems unrelated to child	No. of reported problems	Total
Mobility	2	0	48	50
Self-care	1	1	48	50
Usual activities	4	3	43	43
Pain or discomfort	9	11	30	50
Anxiety or depression	13	8	29	50

Most of the caregivers reported no problems on any of the EQ-5D-3L physical domains (mobility, self-care, usual activities) and fewer reported no problems on the domains of pain/discomfort and anxiety/depression. The problems reported by the caregivers related to having an ill child were mobility – mobility difficulties because of attending to their child 24/7 at the bedside (*n* = 2); self-care – self-care problems owing to being with their child 24/7 and away from home (*n* = 1); usual activities –being in the hospital taking care of their child meant that they were not at home taking care of their other children as they usually would (*n* = 4); pain/discomfort – headaches, back pain and leg pain, also emotional pain of caring for their child in the hospital (*n* = 9); anxiety/depression –worried about the well-being of their child in the hospital and missing the other children at home, worried about financial issues, not being able to go to work and feelings of being overwhelmed (*n* = 13) (Table 4). Problems unrelated to the child were attributed to pre-existing health conditions.

## Discussion

There was a significant correlation between the health of the ill child as reported by the proxy and the health of the caregivers themselves as rated on the VAS over the 3 days. Thus, as the perceived health of the child improved so did the caregiver’s self-reported health. This would support previous research findings that the poor health of a child ‘spills over’ and affects the health of the caregiver (Foster et al. 2010; Gerkensmeyer et al. 2013; Rizk et al. 2011; Zhou et al. 2014). Illness in a loved one has been found to impact one’s health through the increased emotional consequences (Bobinac et al. 2011). This can be seen with a relatively high reporting of problems concerning anxiety/depression and pain and discomfort in the caregivers, which decreases over time as their child’s health improves. The contrary is, however, true for the more physical domains of mobility, self-care and usual activities. The incidence of physical ill health is higher in caregivers with children suffering from a chronic condition owing to the prolonged burden of care (Lavelle et al. 2014; Poley et al. 2011; Tilford et al. 2005; Zhou et al. 2014). The discrepancy between the reporting on physical and emotional/pain dimensions on the second day explains why the latent value of the child and the preference-based scores of the children were not significantly correlated on all 3 days. Furthermore, the increase in problems reported in caregiver’s physical dimensions on the second day could be the cause for the drop in their VAS score on the second day. As 51.5% of the caregivers were staying at their child’s bedside all day, these physical difficulties could be attributed to their discomfort in the hospital where they sleep in a pull-out chair and contribute physically to the care of their child.

Regression analysis showed that the child’s rating of general health, as perceived by the caregiver, significantly influenced the caregiver’s rating of his or her own health on both the first and second days. The child’s proxy VAS score accounted for more of the variance than the caregiver’s reporting of problems in the dimensions on the EQ-5D-3L. Most notably, on the first day of testing, when the child was presumed to be the most ill, the perceived health of the child accounted for 21% of the variance, which was substantially higher than the caregiver’s report of anxiety or depression (6%). These results are similar to those found in a study by Verstraete et al. (2018b), where the perceived health of younger children (aged 1–36 months) significantly influenced the health of the caregiver (Verstraete et al. 2018a). This study further suggests that as the child’s perceived health improves, with a lower VAS score, it contributes to less of the variance in the caregiver’s self-rated health.

The caregivers’ report on day 1 suggests that there are many direct and indirect factors of their child’s ill health which affect their HRQoL. A high number of caregivers reported pain and discomfort, and anxiety and depression as pre-existing conditions. The levels of anxiety and depression are lower than the 34.9% prevalence reported in the peri-urban community of Khayelitsha, Cape Town (Havenaar et al. 2007).

Family-centred policies and services in paediatric healthcare that consider the health of the caregiver can assist in reducing negative influences on the caregiver’s health. Future work should address the extent to which these services would impact CUA on a larger sample of acutely ill children.

## Limitations and recommendations

The results of this study were limited as no adjunct measures were used to measure the severity of the child’s illness or pre-morbid function. Neither was the caregiver’s pre-morbid function or level of stressors considered. This should be explored in future work. It is further recommended that future studies investigate the accuracy of proxy reporting and spill-over effect between the child and the caregiver on a larger age range and sample of children. This will assist in providing clarity if the accuracy of proxy reporting differs according to the child’s age on the same instrument. Furthermore, it could clarify whether the spill-over varies according to age and dependency of the child on the caregiver.

## Conclusion

The strong relationship between the VAS scores of the children and their caregivers, and the influence of the child’s perceived health on the caregiver’s self-reported health, indicates that there is a relationship between the health of the child and the caregiver. Thus, improving the HRQoL of the child will lead to improved HRQoL in the caregiver and should be considered in future societal CUA calculations.
